# Through the Telephone

**Published:** 1897-08

**Authors:** 


### Through the Telephone.

Mrs. Merony: Is that you, doctor?

Doctor: Yes; who is it?

Mrs. Merony: Mrs. Merony. Oh, doctor, what shall I do for the baby? He has swallowed a dime.

Doctor: Well, you surely don't want to spend two dollars to get a dime, do you?

And the telephone ceased to work.—*Newman Indpendent.*

